# Hybrid iterative reconstruction in ultra-low-dose CT for accurate pulmonary nodule assessment: A Phantom study

**DOI:** 10.1097/MD.0000000000041612

**Published:** 2025-02-21

**Authors:** Li-Guo Chen, Hung-Wen Kao, Ping-An Wu, Ming-Huei Sheu, Hsing-Yang Tu, Li-Chuan Huang

**Affiliations:** a Department of Medical Imaging, Hualien Tzu Chi Hospital, Buddhist Tzu Chi Medical Foundation, Hualien, Taiwan; b Department of Radiology, School of Medicine, Tzu Chi University, Hualien, Taiwan; c Department of Medical Imaging and Radiological Sciences, Tzu Chi University, Hualien, Taiwan.

**Keywords:** hybrid iterative reconstruction, iDose^4^ algorithm, noise reduction, Phantom study, pulmonary nodules, solid nodule detection

## Abstract

This study evaluated hybrid iterative reconstruction in ultra-low-dose computed tomography (ULDCT) for solid pulmonary nodule detection. A 256-slice CT machine operating at 120 kVp imaged a chest phantom with 5 mm nodules. The imaging process involved adjusting low-dose computed tomography (LDCT) settings and conducting 3 ULDCT scans (A–C) with varied minimum and maximum mA settings (10/40 mA). Images were processed using iDose^4^ iterative reconstruction at levels 5 to 7. Measurements were taken for noise, signal-to-noise ratio (SNR), contrast-to-noise ratio (CNR), noise power spectrum (NPS), and detectability index (D’) to assess image quality, noise texture, and detectability. Analysis of variance (ANOVA) was used to compare the protocols. Noise levels varied significantly across iDose^4^ iterative reconstruction levels, with the highest noise at 178 HU in iDose^4^ L5 (protocol C) and the lowest at 54.85 HU in level 7 (protocol A). ULDCT scans showed noise increases of 38.5%, 104.2%, and 118.7% for protocols A, B, and C, respectively, compared to LDCT. Protocol A (iDose^4^ level 7) significantly improved SNR and CNR (*P* < .001). The mean volume CT dose index was 2.4 mGy for LDCT and 2.0 mGy, 1.2 mGy, and 0.7 mGy for ULDCT protocols A, B, and C, respectively. Increasing iDose^4^ levels reduced noise magnitude in the NPS and improved the D’. ULDCT with iDose^4^ level 7 provides diagnostically acceptable image quality for solid pulmonary nodule assessment at significantly reduced radiation doses. This approach, supported by advanced metrics like NPS and D’, demonstrates a potential pathway for safer, effective lung cancer screening in high-risk populations. Further clinical studies are needed to validate these findings in diverse patient populations.

## 1. Introduction

Lung cancer is one of the leading causes of death worldwide. Low-dose computed tomography (LDCT) screening has effectively reduced lung cancer mortality in heavy smokers through secondary prevention.^[1–3]^ Pulmonary nodules are the most common chest LDCT finding associated with lung cancer and may require multiple scans for evaluation. However, despite the effectiveness of LDCT in reducing lung cancer mortality, repeated exposure raises concerns about radiation risks, emphasizing the need for ultra-low-dose computed tomography (ULDCT) protocols. Additionally, concerns about radiation exposure arise with lung cancer screening, not only in asymptomatic individuals presenting with lung nodules but also across broader populations undergoing screening. However, repeat CT scans showed a 1.8% excess risk of lung cancer in patients who underwent LDCT (effective dose [ED] approximately 1 mSv) annually starting at the age of 50 to 75 years.^[3]^

Computer tomography scans, although highly effective, may pose a small risk of cancer after prolonged exposure, with some studies quantifying this risk over a period of 10 years.^[4]^ To mitigate this, the “as low as reasonably achievable” (ALARA) principle has been widely adopted, emphasizing radiation dose reduction without compromising diagnostic quality.^[5,6]^ Radiation dose optimization is critical, particularly in screening scenarios, in light of cancer risks associated with CT and guidelines from the National Academies of Sciences Biologic Effects of Ionizing Radiation VII report.^[7]^ Iterative reconstruction (IR) techniques effectively reduce radiation while preserving image quality.^[5,8–10]^ However, reducing radiation dose can increase quantum noise, making it crucial to use IR and filters to maintain diagnostic accuracy.^[5,8–10]^ In lung cancer screening, solid pulmonary nodules must be carefully evaluated for malignancy risk, with imaging used to characterize these lesions better.^[2,11]^

LDCT follows the guidelines set by the American College of Radiology and the Society of Thoracic Radiology, recommending a maximum CT dose index volume (CTDI_vol_) of 3 mGy for standard body size patients.^[1,12,13]^ Multi-detector CT (MDCT) scanners with 16 or more detector rows are recommended for single-breath-hold scanning.^[13,14]^ ULDCT is receiving increasing attention to further reduce radiation exposure, particularly in lung-nodule detection. However, the advantages and limitations of ULDCT regarding image quality, nodule detection rates, and diagnostic accuracy compared to LDCT remain unclear.^[15]^ A lack of standardized ULDCT protocols across different scanners also hinders its consistent clinical application.^[16]^ Further studies are needed to assess the efficacy of ULDCT protocols, ensuring that they provide diagnostic value while minimizing radiation exposure.

Hybrid iterative reconstruction (HIR) algorithm combines statistical noise reduction algorithms with model-based reconstruction techniques. This dual approach minimizes image noise, even at ultra-low-radiation doses, while preserving fine anatomical details critical for accurate diagnosis.^[17]^ By leveraging HIR, our study hypothesizes that ULDCT can achieve clinically acceptable image quality comparable to traditional low-dose protocols, enabling safe and effective imaging in vulnerable populations.

## 2. Materials and methods

### 2.1. Experimental protocols for anthropomorphic phantoms

The commercial RANDO phantom (The Phantom Laboratory, Salem) comprises natural skeletal and plastic tissue-equivalent materials and 36 sections (numbered 0–35). Sections 0 to 34 are 2.5 cm thick, while section 35 (pelvic floor) is approximately 9 cm thick. Sections 0 and 35 contain holes for the placement of dosimeters. Image acquisition was performed using the evaluation phantom with rod inserts simulating photon attenuation in lung-nodule materials. The inserted lung nodules were 5 mm in diameter. The phantom manufacturer supplied nodules with a radiodensity of 20 to 80 HU. A 5-mm nodule was chosen for the study to simulate early-stage lung cancer detection, as it represents the clinically significant threshold for screening. Research indicates that solid nodules under 5 mm carry a malignancy risk of ≤ 1%, aligning with guidelines suggesting minimal to no routine follow-up for low-risk patients.^[18,19]^ This size criterion is vital for early detection, as such small nodules are commonly identified in lung cancer screening, ensuring the study remains focused on clinically relevant scenarios for effective intervention. Ethical approval was not necessary for this study since it involves phantoms.

### 2.2. CT scan protocol

The CT scans were performed using a 256-slice scanner (Brilliance iCT; Philips Healthcare, Best, the Netherlands) with a 128 × 0.625 mm detector and a scanning range of up to 8 cm when the tube rotated around the subject once. The parameters used for LDCT scans with DoseRight automatic exposure control (AEC; Philips Healthcare) were as follows: tube voltage = 120 kVp; tube current was modulated via AEC (DoseRight index min/max = 10/40 mA); scan duration = 0.33 seconds; pitch = 0.758 mm; scan range: the whole lung; scan time = 2.4 seconds; thickness of the reconstructed cross-section = 1.5 mm; and slice interval = 1 mm. The LDCT scans was performed at 120 kVp with AEC tube current. The scans covered the entire phantom with a constant scan length of 40 cm along the *z*-axis, simulating the dimensions of an adult lung.

### 2.3. Image reconstruction

All CT images were reconstructed using iDose^4^. Groups were scanned with reduced exposure settings using HIR algorithm levels. For IR, a prototype of the Philips iDose^4^ system was used for subsequent offline raw data reconstructions to reduce noise in both the raw and image data domains. The iDose^4^ levels ranged from 1 to 7 and determined the strength of the IR algorithm, with higher levels indicating greater noise reduction.^[20]^

All image datasets of the iDose^4^ group were reconstructed at levels 5 to 7 using a slice thickness of 1.5 mm, increments of 1 mm, and a lung convolution kernel.^[11,21,22]^ The reconstructed field of view was 360 mm and the image matrix was 512 × 512 pixels. The iDose^4^ levels (5–7) were chosen to balance image quality and radiation dose reduction, aligning with standard clinical practices. These levels provide sufficient noise reduction for diagnostic accuracy while minimizing artifacts and preserving anatomical details, which is critical for lung-nodule evaluation.

### 2.4. Radiation dose assessment

The radiation dose of the LDCT protocols was estimated based on the CTDI_vol_ and the dose-length product (DLP; Eq. 1). The results of the measures were displayed and recorded on the scanner.


$$\begin{array}{l} DLP\;\;\;\left(mGy\cdot cm\right)=CTDI_{vol}\;\;\;\left(mGy\right)\cdot scan\;\;\;length\;\;\;\left(cm\right) \end{array}$$ (1)

The ED was calculated by multiplying the DLP by the dose conversion factor *k* (0.014 mSv mGy^−1^cm^−1^; Eq. 2), as recommended by the European Guidelines for Multislice CT.^[23]^


$$\begin{array}{l} ED\;\;\;\left(mSv\right)=DLP\;\;\;\left(mGy\cdot cm\right)\;\;\;\times \;\;\;k \end{array}$$ (2)

### 2.5. Image quality assessment

For quantitative analysis, the image quality of LDCT of the middle lobes of the lungs (window width/level = 1200/−500) was assessed using Image J (version 1.51p).^[24]^ We assessed Image noise, contrast-to-noise ratio (CNR), and signal-to-noise ratio (SNR) for each CT image series.^[22,25–27]^ Circular regions of interest were placed in the lung (10 mm^2^) and lung nodules (10 mm^2^) to measure and record the CT values. The mean image noise was defined as the average of the standard deviation (SD) of the attenuation in 6 consecutive ROI measurements at different slice positions. Two CT values and their standard deviations were recorded. Subsequently, SNR and CNR were calculated using equations 3 and 4. Additionally, we analyzed the noise texture using the noise power spectrum (NPS) and assessed low-contrast detectability and image quality through the detectability index (D’).


$$\begin{array}{l} SNR=\frac{\#CT_{nodules\;\;\;}\left(HU\right)}{SD_{lung}\left(HU\right)} \end{array}$$ (3)

$$\begin{array}{l} CNR=\frac{\left|\#CT_{lung}\;\;\;\left(HU\right)\;\;\;-\;\;\;\#CT_{nodules}\left(HU\right)\right|}{SD_{lung}\;\;\;\left(HU\right)} \end{array}$$ (4)

### 2.6. Statistical analysis

Statistical power analysis was conducted using G*Power to determine the appropriate sample size for the study. Based on the anticipated effect size and desired alpha level (e.g., 0.05), the required power was set at 0.8, standard in many statistical tests to minimize the likelihood of Type II errors. This analysis confirmed that our sample size was sufficient to detect meaningful outcome differences. We performed a 1-way analysis of variance (ANOVA) in GraphPad Prism (version 9; GraphPad Software, San Diego) to assess whether significant differences (*P* < .05) in CTDI_vol_, SNR, and CNR existed between phantom images acquired with ULDCT at varying levels of IR (protocols A, B, and C) and those obtained with LDCT.

## 3. Results

### 3.1. Radiation dose index in ULDCT and LDCT

The dose index of CTDI_vol_ in LDCT is 2.4 mGy, while ULD protocols range from 0.7 to 2 mGy. DLP in LDCT is 95.8 mGy·cm, whereas ULD protocols range from 27.9 to 79.8 mGy·cm. The ED in LDCT is 1.34 mSv, and for ULD protocols, it ranges from 0.39 to 1.34 mSv. Dose reduction in ULDCT protocols ranges from 16.7% to 70.1% compared to LDCT (Table 1). The dose index for ULD protocols A, B, and C, as well as the low-dose protocols and dose reduction of ULD protocols compared to LDCT, are depicted in Table 1.

**Table 1:** Scan protocols and radiation dose index.

Protocol	Scans	AEC (mA)	CTDI_vol_ (mGy)	DLP (mGy-cm)	Effective dose (mSv)	Dose reduction (%)
Low-dose	Control	32 to 40	2.4	95.8	1.34	-
ULD	A	25 to 32	2	79.8	1.12	16.7
	B	13 to 16	1	39.9	0.56	58.3
	C	10	0.7	27.9	0.39	70.1

AEC = automatic exposure control, CTDI_vol_ = CT volume dose index, DLP = dose-length product, ULD = ultra-low-dose.

### 3.2. Image quality index of SNR, CNR, and noise

**Figure 1.**
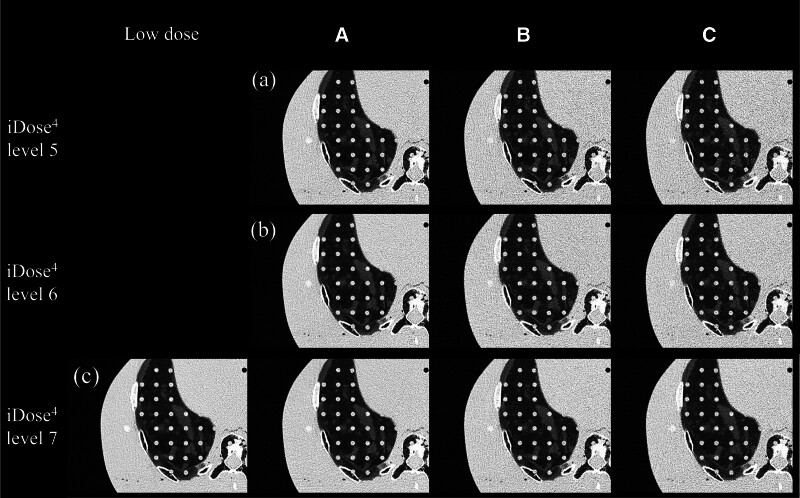
A 5-mm solid nodule in the middle lobes of the lungs was reconstructed with the iDose^4^ IR technique at strength levels of 5 to 7. (A) iDose^4^ level 5; (B) iDose^4^ level 6; (C) iDose^4^ level 7.

**Figure 2.**
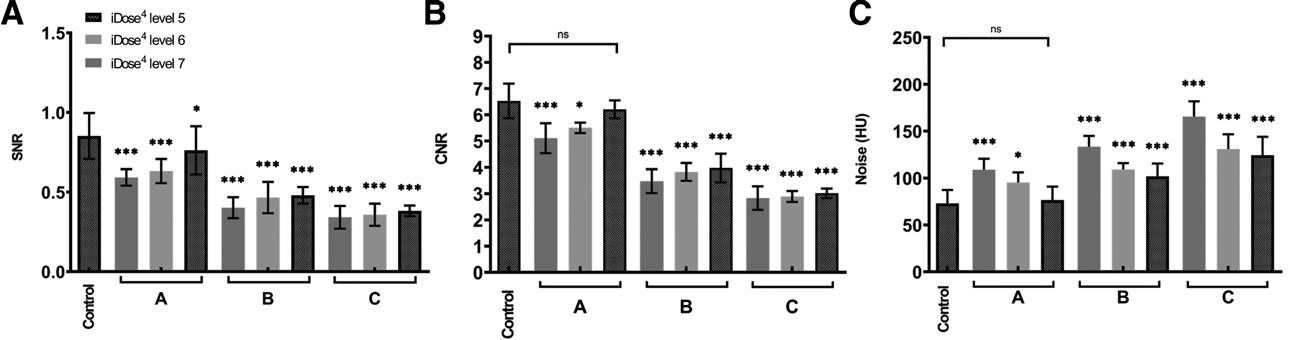
Computed tomography image quality index analyses reconstruction techniques in 3 protocols. (A) SNR, (B) CNR, and (C) Noise. CNR = contrast-to-noise ratio, SNR = signal-to-noise ratio.

Images acquired using different protocols (protocols A, B, and C, as well as the low-dose protocol) in the middle lobes of the lungs were reconstructed using the iDose^4^ IR technique at strength levels of 5 to 7, clearly revealing a 5-mm solid nodule upon visual inspection (Fig. 1). The SNR was the highest in the images acquired with ULD protocol A (2 mGy) reconstructed with iDose^4^ level 7 (mean ± SD: 0.76 ± 0.24) and the lowest in the images acquired with ULD protocol C (0.7 mGy) reconstructed with iDose^4^ level 7 (mean ± SD: 0.42 ± 0.19). Comparison of the image SNRs among iDose^4^ levels 5 to 7 revealed that the SNRs of protocols B and C differed significantly from those of LDCT, while that of protocol A with iDose^4^ level 7 did not (Fig. 2A).

The CNR was highest in the images acquired with ULD protocol A (2 mGy) reconstructed with iDose^4^ level 7 (mean: 6.58 ± 0.26) and the lowest those acquired with ULD protocol C (0.7 mGy) reconstructed with iDose^4^ level 7 (mean: 3.21 ± 0.21). The CNR of iDose^4^ level 7 did not differ significantly between ULD protocol A and LDCT (Fig. 2B).

The noise level was highest in images acquired with ULD protocol C (0.7 mGy) images reconstructed with iDose^4^ level 5 (mean: 178.95 ± 2.28 HU), while the lowest noise level was acquired with protocol ULD protocol A (2 mGy) images reconstructed with iDose^4^ level 7 (mean: 76.00 ± 2.13). The increase in noise for protocols A through C was compared to that of LDCT to compare image noise using the IR algorithm among iDose^4^ levels 5 to 7. The image noise was significantly higher at levels 5 and 6 than LDCT (*P* < .001). Noise at level 7 did not differ significantly from that of LDCT (Fig. 2C).

### 3.3. NPS of noise texture and D’

**Figure 3.**
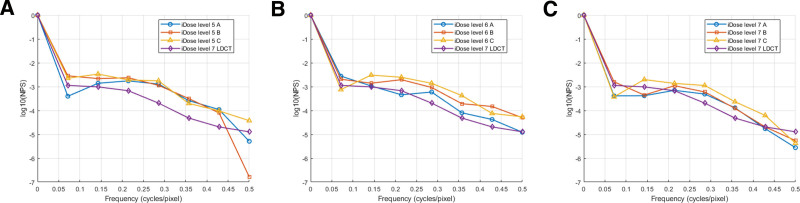
The noise power spectrum, illustrating the intensity and distribution of noise at different spatial frequencies in various protocols. (A) iDose^4^ Level 5, (B) iDose^4^ Level 6, and (C) iDose^4^ Level 7. LDCT = low-dose computed tomography.

**Figure 4.**
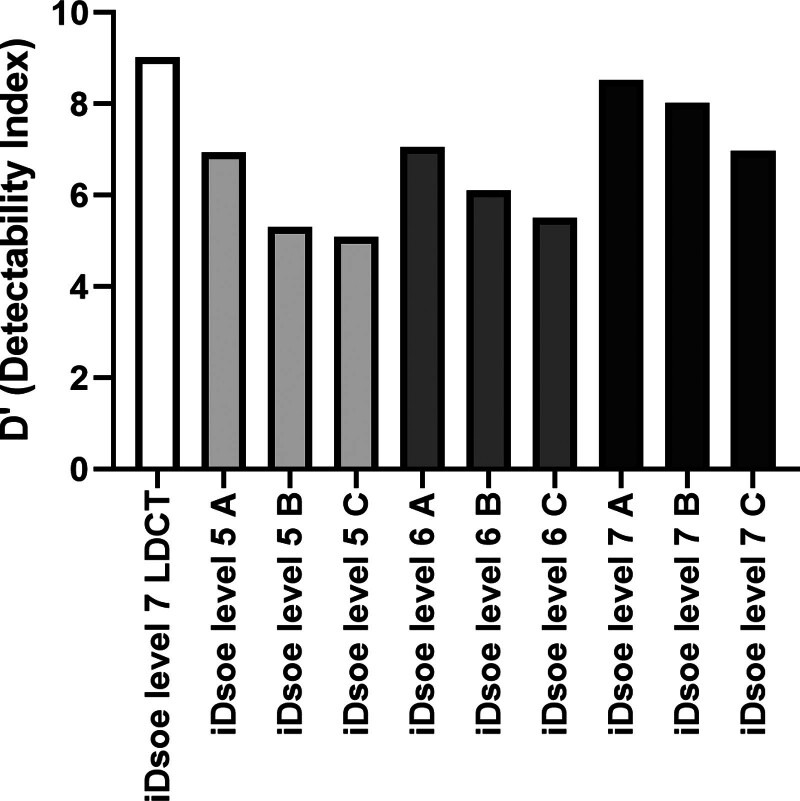
Detectability index (D’) values for different iDose^4^ conditions (levels 5 to 7) in ULDCT and LDCT. LDCT = low-dose computed tomography, ULDCT = ultra-low-dose computed tomography.

The NPS analysis revealed that protocol A (2 mGy) reconstructed with iDose^4^ level 7 exhibited the lowest NPS values in the low-frequency range (0.1–0.3 cycles/pixel), which are critical for preserving diagnostic image clarity. In contrast, protocol C (0.7 mGy) showed significantly higher NPS values in the same frequency range, indicating increased image noise. The differences in NPS values between protocols A, B, and C were statistically significant (*P* < .001) (Fig. 3). The D’ was highest for protocol A (2 mGy) with iDose^4^ level 7, achieving a value of 8.53, indicating superior nodule detectability. Protocols B and C demonstrated significantly lower D’ values, reflecting reduced lesion detection performance as radiation dose decreased. The D’ values for protocol A did not differ significantly from those of LDCT, suggesting that this protocol effectively balances dose reduction with diagnostic performance (Fig. 4). Moreover, increasing iDose^4^ levels progressively improved detectability, with D’ values rising from lower to higher reconstruction strengths.

## 4. Discussion

Optimizing the radiation dose in CT imaging is a continuous endeavor, and this phantom study is the first to evaluate image quality across radiation doses for analyzing solid nodules using ULDCT. Our results showed that the radiation dose can be lowered to 2 mGy of CTDI_vol_ while maintaining the diagnostic quality for evaluating solid pulmonary nodules by combining single-energy CT with an IR algorithm. This study assessed phantom radiation exposure related to image quality by applying the iDose^4^ IR algorithm to lung ULDCT data. The results demonstrate that protocol A reconstruction using iDose^4^ level 7 yielded an overall reduction of 16.7% in CTDI_vol_ compared to LDCT while maintaining image quality for diagnosis. iDose^4^ levels 5 to 6 of protocols B and C, with objective dose measurements, reduced CTDI_vol_ by 58.3% and 70.1%, respectively; however, the image quality was insufficient for diagnosis.

Increasing scan mAs and iDose levels can reduced overall image noise and higher lesion detectability.

### 4.1. Effects of dose reduction on CT image quality

Reducing the radiation dose in CT is usually accompanied by increased quantum noise, which decreases image quality.^[28]^ Some studies have shown that IR significantly reduces image noise and false lesion detection, enhancing the evaluation of solid lung nodules.^[29–31]^ The imaging quality of pulmonary nodules is influenced by image acquisition and reconstruction, software performance, and nodule quantification features.^[32]^ Another study showed that images of ground-glass (4 mm) nodules had superior quality, and spectral photon-counting using iDose^4^ had superior detection ability for solid nodules over dual-layer CT.^[33]^ Studies also found that the lung-nodule detection rate with low-dose IR is the same as conventional dose-filtered back projection (FBP).^[34,35]^ Other phantom studies have shown that IR can accurately determine lung nodule volume, even at low doses.^[33,34]^ Our study indicates that lung nodules can still be detected at a 2 mGy CTDI_vol_ dose level without significantly reducing noise and CNR.

### 4.2. Image quality indices (SNR, CNR, and noise) in LDCT and ULDCT

CNR and SNR are image quality indices: a high SNR is necessary for detecting small lesions and essential for distinguishing between lesions and background tissue. Maintaining high CNR and SNR at low-radiation doses is essential for maintaining diagnostic image quality. Our study found that the CNR was significantly higher in LDCT (7.38 ± 0.70) than ULDCT (6.58 ± 0.26). SNR was also higher for LDCT while evaluating pulmonary nodules (0.91 ± 0.66 vs 0.76 ± 0.24 in ULDCT), but the difference was not statistically significant. We found that image noise increased by up to 38.5%, SNR decreased by 15.4%, and CNR decreased by 10.8% in ULDCT images obtained using protocol A (2 mGy) with the iDose^4^ level 7 algorithm compared to LDCT. However, these differences were insignificant, and LDCT still provided images of good diagnostic quality while reducing the radiation dose. Additionally, LDCT has been found to have high diagnostic accuracy for detecting honeycombing and bronchiectasis, while ULDCT has high diagnostic accuracy for detecting pneumothorax, consolidations, and ground-glass opacities.^[35]^ Determining the diagnostic accuracy of LDCT and ULDCT for other chest pathologies was impossible due to varying outcome measures, lack of precision estimates, and heterogeneous study designs and methodologies.^[35]^

### 4.3. Noise texture and D’ in LDCT and ULDCT

Our results demonstrated that protocol A with iDose^4^ level 7 achieved the lowest NPS values in the low-frequency range (0.1–0.3 cycles/pixel), which is critical for maintaining diagnostic clarity.^[36]^ Increasing the iDose^4^ level reduces overall image noise, particularly at higher spatial frequencies, resulting in enhanced noise suppression with higher IR. While higher IR levels improve the CNR, they may also introduce trade-offs, such as losing fine spatial detail or smoothing effects.

LDCT consistently achieves higher D’ values than ULDCT at similar iDose^4^ levels, reflecting better lesion detectability due to the higher radiation dose. However, as iDose^4^ levels increase, the performance gap between ULDCT and LDCT narrows, especially at higher iDose^4^ strengths (e.g., iDose^4^ level 7; Fig. 3). ULDCT with higher iDose^4^ levels provides acceptable diagnostic performance, making it a viable low-radiation alternative in specific scenarios. These findings highlight the potential of IR techniques to optimize image quality and balance diagnostic accuracy with patient safety, even at ultra-low doses.

### 4.4. IR algorithm and dose index

Our study maintained diagnostic image quality in ULDCT using the iDose^4^ IR algorithm at a radiation dose of 1.12 mSv in protocol A. However, lowering the radiation dose to 0.39 mSv in protocol C resulted in insufficient image quality for diagnosis. We found that using the iDose^4^ level 7 algorithm yielded a 16.7% reduction in CTDI_vol_ compared to LDCT. Using level 5 to 6 algorithms in protocols B and C resulted in worse objective measurements than those at level 7 in protocol A. Our results are consistent with previous studies on iDose^4^ and other IR algorithms.^[34,37,38]^ Notably, our study achieved a reduction of 25.9% in CTDI_vol_ (2 mGy) compared to the low-dose (2.7 mGy) setting used by Kim et al (2018), highlighting the potential for further dose optimization while maintaining acceptable image quality.^[37]^

The National Lung Screening Trial estimated that the mean ED of the LDCT protocol is 1.5 mSv.^[39]^ Studies have reported doses ranging from 0.71 to 2.7 mSv depending on the kV and mA values.^[15,40]^ Thus, the results of the current and previous studies should be compared carefully due to the different methodologies. We estimated the dose using CTDI_vol_ as an alternative, which other researchers can further reproduce. In contrast, our phantom study yielded a significantly lower CTDI_vol_ (2 mGy) while maintaining a high evaluation ability for nodule entities.

The iDose^4^ algorithm balances noise reduction and image quality at various dose levels. Compared to traditional FBP, iDose^4^ achieves significant noise reduction and improves image quality, especially in low-dose protocols, with DLP reductions averaging 28.9% in some studies.^[41]^ However, it offers less noise reduction than advanced algorithms like iterative model reconstruction (IMR), which can reduce noise by up to 88%, while iDose^4^ achieves approximately 72% in similar conditions.^[42]^

However, iDose^4^ is not without limitations. Higher noise reduction levels may affect spatial resolution, which can compromise the detection of smaller or subtle pulmonary nodules. Studies comparing iDose^4^ to other algorithms, such as IMR (Model-Based IR), show that while iDose^4^ effectively reduces noise, IMR may offer superior image quality at similar dose levels.

### 4.5. ULDCT with IR in lung cancer screening or follow-up context

The potential adoption of ULDCT with IR in lung cancer screening and follow-up holds significant promise, particularly in reducing radiation exposure while maintaining image quality. ULDCT with IR, such as the iDose^4^ algorithm, has been shown to reduce radiation dose effectively while retaining diagnostic image clarity, which is crucial in lung cancer screening programs that require frequent imaging.

In the context of lung cancer screening, adopting ULDCT could supplement or, in some cases, replace existing low-dose protocols. The reduced dose levels and the noise reduction capabilities of IR algorithms enable high-quality imaging with minimal radiation exposure, aligning well with current guidelines prioritizing dose minimization in screening programs. However, while ULDCT with IR shows promise, its ability to detect smaller lesions with similar accuracy to conventional low-dose CT needs further validation in large-scale studies to ensure that it does not compromise sensitivity.

Ultimately, ULDCT with IR may not completely replace current low-dose CT protocols but could serve as an alternative or complementary approach, particularly for individuals at lower risk or for follow-up imaging where frequent scans are needed.

This study has some limitations. First, the phantom only represented a standard-sized adult, which limits the generalization of the results to heterogeneous body types.^[34]^ Factors such as patient age, body mass index, and lung tissue density may significantly impact image quality and noise levels, necessitating further validation in diverse patient populations.^[34]^ Second, we only investigated solid nodules and did not differentiate further between subsolid and ground-glass nodules. Finally, changing the tube voltage between the low- and ultra-low-dose protocols could have influenced the conspicuity of the lesions. Future studies should aim to change only the tube current and IR. Future research should validate these findings in human trials, including populations with diverse body types and pulmonary nodule characteristics. Studies incorporating various nodule types and sizes and long-term investigations into the periodic use of ULDCT with iDose^4^ are necessary to establish its utility in lung cancer screening.

## 5. Conclusions

Our study demonstrates that HIR in ULDCT maintains high diagnostic accuracy for pulmonary nodule assessment while significantly reducing radiation exposure. These results align with guidelines advocating for low-dose CT in populations requiring repeated imaging, such as those at high risk for lung cancer, younger patients, and individuals with chronic conditions requiring follow-up. Clinically, integrating HIR with ULDCT could enhance the early detection and monitoring of pulmonary nodules without increasing radiation risks. This adaptation could be incorporated into existing guidelines, such as those from the Fleischner Society, to emphasize IR techniques for routine use in vulnerable populations.

## Acknowledgments

We thank the Department of Radiation Oncology for lending us the study phantom.

## Author contributions

**Data curation:** Li-Guo Chen.

**Formal analysis:** Li-Guo Chen, Li-Chuan Huang.

**Investigation:** Li-Guo Chen, Li-Chuan Huang.

**Methodology:** Li-Guo Chen, Hung-Wen Kao, Li-Chuan Huang.

**Project administration:** Li-Guo Chen, Li-Chuan Huang.

**Supervision:** Hung-Wen Kao, Ping-An Wu, Ming-Huei Sheu, Hsing-Yang Tu, Li-Chuan Huang.

**Validation:** Li-Guo Chen, Li-Chuan Huang.

**Visualization:** Li-Guo Chen, Li-Chuan Huang.

**Writing—original draft:** Li-Guo Chen, Li-Chuan Huang.

**Writing – review & editing:** Hung-Wen Kao, Ping-An Wu, Ming-Huei Sheu, Hsing-Yang Tu, Li-Chuan Huang.
